# Facts and Figures from the Annual Reports

**Published:** 1909-10-23

**Authors:** 


### FACTS AND FIGURES FROM THE ANNUAL REPORTS.

### THE ABUSE OF HOSPITALS.

Attention is drawn in the recently issued annual report of the West Bromwich District Hospital to the increasing abuse by patients able to pay for advice of hospital privileges. Many people suffering from trivial complaints attended as out-patients, and help to account for the large increase in that department. The committee considers that a curtailment in the number of notes issued would be to the interest of the hospital.

The hospital, it is interesting to note, now presents its accounts on the modified uniform system adopted by the King Edward's Hospital Fund and the Hospital Sunday and Saturday Funds for London, which has necessitated an extensive re-arrangement of the various items of finance.

The income of the hospital does not increase in proportion to the largely increased work the institution is now called upon to perform, and unless further financial help is immediately forthcoming the board must take into serious consideration the question of closing a ward, or possibly the out-patient department (as has been done in other similar institutions).

Mr. E. J. Hunt has been elected to fill the vacancy on the board caused by the death of Alderman Williams, and Mr. S. Withers has intimated his intention not to seek re-election to the board, having left the district.

The hospital has received very handsome gifts of aseptic furniture, bed lockers, and appliances, to the value of £500, from the President, Mr. F. Dudley Docker, and aseptic dressing tables and wheel chairs to the value of £100 from Colonel G. Walton Walker.—*West Bromwich District Hospital Annual Report.*

